# A quantitative analysis of stem cell homeostasis in the *Arabidopsis* columella root cap

**DOI:** 10.3389/fpls.2015.00206

**Published:** 2015-03-27

**Authors:** Jing Han Hong, Huangwei Chu, Chen Zhang, Dipanjana Ghosh, Ximing Gong, Jian Xu

**Affiliations:** ^1^Department of Biological Sciences, National University of Singapore, Singapore; ^2^NUS Centre for BioImaging Sciences, National University of Singapore, Singapore

**Keywords:** *Arabidopsis*, columella root cap, EdU staining, Lugol’s staining, stem cell homeostasis

## Abstract

The Lugol’s staining method has been widely used to detect changes in the maintenance of stem cell fate in the columella root cap of *Arabidopsis* roots since the late 1990s. However, various limitations of this method demand for additional or complementary new approaches. For instance, it is unable to reveal the division rate of columella root cap stem cells. Here we report that, by labeling dividing stem cells with 5-ethynyl-2′-deoxyuridine (EdU), the number and distribution of their labeled progeny can be studied so that the division rate of stem cells can be measured quantitatively and in addition, that the progression of stem cell progeny differentiation can be assessed in combination with Lugol’s staining. EdU staining takes few hours and visualization of the stain characteristics of columella root cap can be performed easily under confocal microscopes. This simple technology, when used together with Lugol’s staining, provides a novel quantitative method to study the dynamics of stem cell behavior that govern homeostasis in the *Arabidopsis* columella root cap.

## Introduction

In multicellular organisms, a pool of stem cells is maintained throughout the life cycle to produce exactly and only as many cells as necessary for a specific tissue, an astonishing natural phenomenon known as stem cell homeostasis. Stem cells divide asymmetrically to produce two daughter cells of different fates. One of the daughters retains the self-renewal properties of the mother stem cell and keeps on dividing, whereas the other daughter is programmed to differentiate into a specialized mature cell. Asymmetrical division of stem cells and differentiation of mature cells are dynamically regulated so as to maintain the balance between self-renewal and lineage commitment in response to tissue requirements. In animals altered stem cell homeostasis has been linked to aging and carcinogenesis in various organs (Tieu et al., 2012; Signer and Morrison, 2013; Oh et al., 2014; Vermeulen and Snippert, 2014); whereas in plants the longevity of the shoot apical meristem of trees reflects a well-maintained stem cell homeostasis that can last for over 1000 years (Doerner, 2003; Perales and Reddy, 2012).

To reveal the mechanisms that regulate stem cell homeostasis, the dynamics of stem cell behaviors have been studied in various animal tissues where rapid cell renewal is needed. Because the intestine is one of the most rapidly regenerated tissues in the body, the intestinal crypt has become the archetypal system for quantitative understanding of stem cell dynamics and regulation (van der Flier and Clevers, 2009; Clevers, 2013; Ritsma et al., 2014). Several labeling methods have been developed to mark intestinal stem cells that are undergoing cell division. For instance, BrDU (5-bromo-2′-deoxyuridine), titrated thymidine and increasingly 5-ethynyl-2′-deoxyuridine (EdU) have been used to evaluate the division rate of intestinal stem cells (Salic and Mitchison, 2008; Yin et al., 2014) and to test their response to DNA damage (Potten et al., 2002; Hua et al., 2012; Zhu et al., 2013). In roots of plants, EdU staining has also been employed to investigate the division rates of meristem and quiescent center (QC) cells (Vanstraelen et al., 2009; Kotogány et al., 2010), but its use on the analysis of rate of stem cell division has not been reported.

**FIGURE 1:**
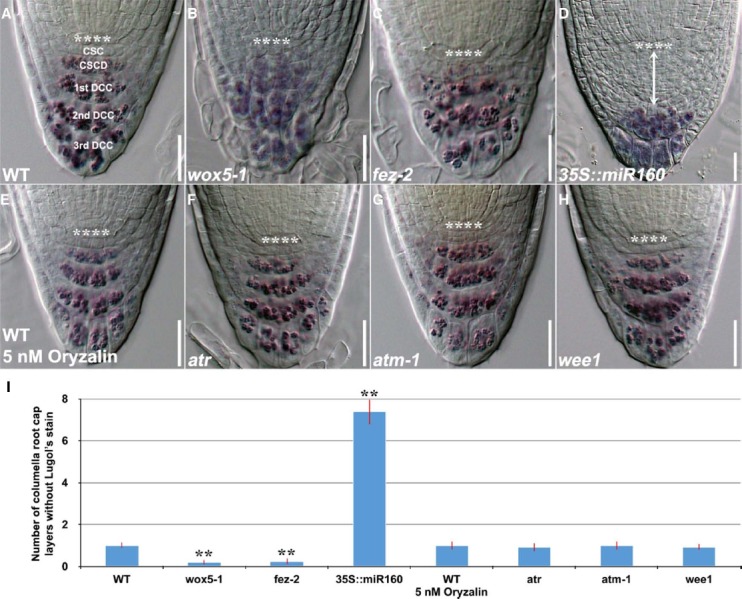
**Lugol’s staining showing accumulation patterns of starch granules in the columella root cap of 4-day-old WT and mutant genotypes. (A)** WT (*n* = 14). **(B)** wox5-1 (*n* = 15). **(C)** fez-2 (*n* = 11). **(D)** 35S::miR160 (*n* = 15). **(E)** WT treated with 5 nM oryzalin for 24 h (*n* = 11). **(F)** atr (*n* = 12). **(G)** atm-1 (*n* = 12) and **(H)** wee1 (*n* = 12). **(I)** Quantification of the number of layers of unstained columella root cap cells. Error bars represent standard error of the mean. **: *P* < 0.01, *t*-test. **** represents position of the QC. CSC: layer of columella root cap stem cells; CSCD: layer of differentiating columella root cap stem cell daughters; 1st DCC: 1st layer of fully differentiated columella root cap cells; 2nd DCC: 2nd layer of fully differentiated columella root cap cells; 3rd DCC: 3rd layer of fully differentiated columella root cap cells. Scale bars: 20 μm.

In plants, regulation of stem cell homeostasis has been studied in specialized regions known as meristems, which contain the stem cells. The most important meristems, the shoot apical meristem and root meristem, are located at the tips of shoots and roots and responsible for almost all the growth that occurs post-embryonically. The root meristem, but not the shoot apical meristem, contains stem cells that are responsible for maintaining a specific tissue and as such resemble animal stem cells. The columella root cap, a terminal tissue of roots of most plants, can be seen as an analog to the intestine, although with a simpler tissue organization (Heidstra and Sabatini, 2014). A typical columella root cap in the dicot model plant *Arabidopsis thaliana* (*Arabidopsis*) consists of one layer of columella root cap stem cells resided below the mitotically less-active QC, one layer of differentiating columella root cap stem cell daughters and 3 layers of fully differentiated columella root cap cells (Figure 1A). Columella root cap stem cells divide asymmetrically to produce two daughters: one retains stem cell properties and the other, the columella root cap stem cell daughter, is committed to differentiate, without further round of division, into columella root cap cells that will ultimately detach from the root (Bennett and Scheres, 2010; Bennett et al., 2014). Under physiological conditions, QC cells were found to divide infrequently to replace the columella root cap stem cells and thus populate the columella root cap region (Cruz-Ramirez et al., 2013).

Both differentiating columella root cap stem cell daughters and fully differentiated columella root cap cells contain starch granules that are not found in columella root cap stem cells, leading to the use of Lugol’s staining as a rapid method for the detection of cell fate in the *Arabidopsis* columella root cap (van den Berg et al., 1997). Columella root cap stem cells do not contain starch granules and thus will not be stained as the rest of the columella root cap (Figure 1A). But this static observation do not necessarily imply either extra division, failure to differentiate or more cells with stem cell-like identity (Bennett et al., 2014). Thus, the Lugol’s staining method is incapable of quantitatively assessing the rate of stem cell division and the progression of stem cell progeny differentiation in the columella root cap. Furthermore, this method may not be applicable to mutants that are defective in starch metabolism.

To permit quantitative analysis of stem cell homeostasis in the columella root cap, in this article, we propose a simple method to examine the rate of stem cell division and the progression of stem cell progeny differentiation in the *Arabidopsis* columella root cap. EdU, a nucleoside analog of thymidine, can be incorporated into the newly synthesized DNA strand (Salic and Mitchison, 2008). An Alexa Fluor^®^ dye, containing an azide side chain, reacts with the alkyne group in the EdU in a click reaction. This allows for the visualization of the labeled DNA in cells undergoing cell division during the period of EdU staining under a fluorescent microscope (Kolb et al., 2001; Rostovtsev et al., 2002; Breinbauer and Kohn, 2003; Wang et al., 2003), thus permitting the quantification of rate of stem cell division in the columellar root cap. In addition, the distribution of EdU-labeled stem cell progeny can be revealed which, when used together with Lugol’s staining, providing a methodology for the assessment of progression of stem cell progeny differentiation.

## Materials and Methods

### Plant Materials and Growth Conditions

*wox5-1* (van den Berg et al., 1997), *fez-2* (Willemsen et al., 2008), and *35S::miR160* (Wang et al., 2005; Ding and Friml, 2010) were described previously. *atr* (SALK_054383) and *wee1* (SALK_147968C) were obtained from the Nottingham *Arabidopsis* Stock Centre (NASC). *atm-1* is a kind gift from Lieven De Veylder’s lab (De Schutter et al., 2007). The seeds were sterilized with 15% (v/v) bleach solution for 20 minutes and washed 3 times with autoclaved milli-Q water before stratification at 4°C for 48 h in the dark. Seeds were sown on ½ Murashige and Skoog (MS) medium with 0.8% agar and allowed to grow vertically.

### EdU Staining

After 3 days of germination, the seedlings were transferred to on ½ MS medium with 0.8% agar supplemented with 10 μM EdU (Invitrogen Click-iT^®^ EdU Imaging Kit) and allowed to grow vertically for 24 h. The seedlings were then fixed in freshly prepared fixative solution containing 3.7% (v/v) paraformaldehyde (Sigma) and 1% (v/v) Triton-X 100 (Sigma) in 1 × PBS solution (Vivantis) for 1 h in a vacuum chamber and washed twice with 3% (w/v) bovine serium albumin (BSA; Santa Cruz) in 1 × PBS solution. The seedlings were then incubated with 50 μl Click-iT^®^ reaction cocktail (Invitrogen Click-iT^®^ EdU Imaging Kit C10340; 43 μl of 1 × Click-iT^®^ EdU reaction buffer, 2 μl of CuSO_4_, 0.12 μl of Alexa Fluor^®^ azide and 5 μl of 1 × Click-iT^®^ EdU buffer additive) for 1 h protected from light at room temperature. The stained seedlings were then washed once with 3% (w/v) BSA in 1 × PBS solution and then stored in 1 × PBS solution protected from light until imaging.

### Imaging for EdU Stained Samples

The EdU stained seedlings were mounted in 1 × PBS solution and imaged using Leica TCS SP5X confocal microscopy with Leica HCX PL APO 63×/1.20W CORR CS lens. The excitation wavelength used was 647 nm (white light laser) and emission wavelengths between 655 and 750 nm were collected. Differential interference contrast (DIC) images of the seedlings were also captured in addition to the EdU stain.

### Lugol Staining

The roots of the seedlings were submerged in Lugol solution (Sigma) for 30 s. The roots were then mounted onto microscopic slides using clearing buffer (chloral hydrate:glycerol:water in 8:3:1 ratio) as the mounting medium. The seedlings were imaged using a DIC optic on a LEICA CTR5000 DIC microscope equipped with a Nikon DS-Ri1 Camera.

## Results

### EdU Staining Reveals Disrupted Stem Cell Homeostasis in Mutants with Defects in the Columella Root Cap

Using Lugol’s staining technology, accumulation of starch granules at the position of columella root cap stem cells was found after QC ablation (van den Berg et al., 1997) and in *wox5-1* mutants (Figure 1B), resulting in the conclusion that the QC and WOX5 inhibit differentiation of columella root cap stem cells (van den Berg et al., 1997; Sarkar et al., 2007; Ding and Friml, 2010). A similar phenotype pattern in the *fez-2* mutant (Figure 1C) was however, attributed to decreased cell division (Willemsen et al., 2008). Likewise, *miR160C* overexpression plants (*35S::miR160*; Figures 1D–I) were shown to have more layers of columella root cap cells without starch granules (Wang et al., 2005; Ding and Friml, 2010). However, as previously discussed, this static method is unable to elucidate the columella stem cell homeostasis.

In our method, we transfer the *Arabidopsis* seedlings to MS agar plates supplemented with EdU for up to 24 h to allow columella root cap cells to divide and differentiate. Of all the columella root cap cells, only the columella root cap stem cells, that have the ability to asymmetrically divide, will acquire the EdU stain. As the columella root cap stem cells divide continuously, differentiating stem cell progeny that also have the EdU stain constantly “moves” down the axis toward the tip of the root cap. The faster the division and differentiation, the more the EdU stain can be detected at lower columella root cap layers.

**FIGURE 2:**
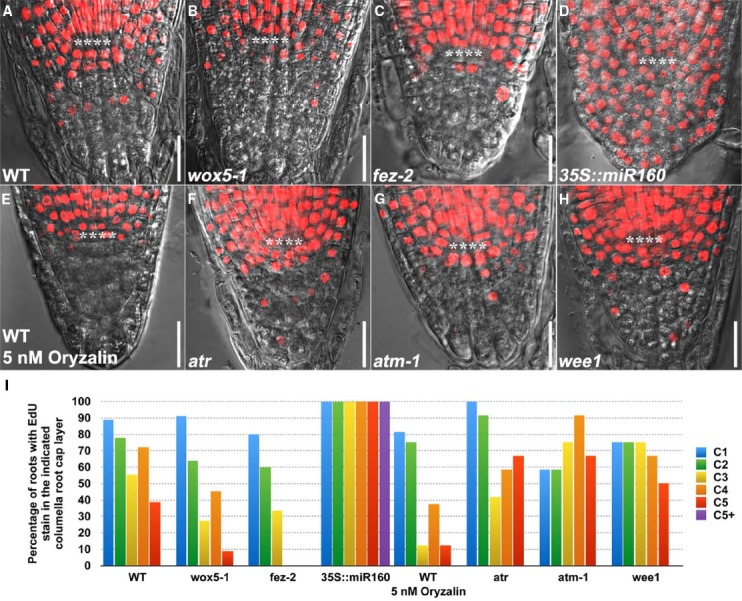
**EdU Staining patterns in the columella root cap of 4-day-old WT and mutant genotypes. (A)** WT (*n* = 18). **(B)** wox5-1 (*n* = 11). **(C)** fez-2 (*n* = 15). **(D)** 35S::miR160 (*n* = 10). **(E)** WT treated with 5 nM oryzalin (*n* = 16) for 24 h. **(F)** atr (*n* = 12). **(G)** atm-1 (*n* = 12) and **(H)** wee1 (*n* = 12). All WT and mutant seedlings were treated with 10 μM EdU for 24 h before imaging. **(I)** Quantification of the percentage of roots with EdU stain in the indicated columella root cap layer. **** represents position of the QC. C1: 1st columella root cap layer below the QC, corresponding to the layer of columella root cap stem cells (CSC); C2: 2nd columella root cap layer below the QC, corresponding to the layer of differentiating columella root cap stem cell daughters (CSCD); C3 to C5: 3rd to 5th columella root cap layers below the QC, corresponding to 1st to 3rd layers of fully differentiated columella root cap cells (1st to 3rd DCCs). C5^+^: extra columella root cap layer(s) in 35S::miR160. Note that cell fate in the columella root cap may be altered in the mutant genotypes. Scale bars: 20 μm.

We observed that *wox5-1* (Figures 2B,I) and *fez-2* (Figures 2C,I) had decreased percentage of roots with EdU stain at lower columella root cap layers as compared to the wild-type (WT) control (Figures 2A,I), suggesting that columella root cap stem cells were still present in *wox5-1* and *fez-2* but their division rates were decreased. By contrast, the division rate of columella root cap stem cells in *35S::miR160* appeared to be enhanced (Figures 2D,I) by overexpression of *miR160C*, leading to the EdU stain in almost all the columella root cap cells (Figure 2D). Notably, Lugol’s stain could be seen in cells of lower columella root cap layers (Figure 1D), indicating occurrence of cell differentiation. Thus, overexpression of *miR160C* led to an imbalance between stem cell self-renewal and commitment to differentiation.

### EdU Staining Reveals Alterations in Stem Cell Behavior in the Columella Root Cap that Failed to be Identified by Lugol’s Staining

We further validated the robustness of our method by chemical and genetic perturbation of cell division. After treatment with oryzalin, a microtubule assembly inhibitor that induces cell cycle arrest at G2/M phase (Ehler and Dutcher, 1998), fewer roots were observed to have EdU stain in the lower columella root cap layers (Figures 2E,I), thus confirming the inhibitory effect of oryzalin on cell division and the sensitivity of EdU staining in detecting changes in cell division. By contrast, Lugol’s staining failed to detect any changes (Figure 1E), indicating that division rather than differentiation was altered by oryzalin treatment.

Similarly, while no obvious changes were found with Lugol’s staining (Figures 1F–I), EdU staining revealed that mutations in DNA damage-response components such as ATR, ATM, and WEE1, which activate a transient cell cycle arrest that allows cells to repair damaged DNA before proceeding into mitosis (Sancar et al., 2004; De Schutter et al., 2007; De Veylder et al., 2007; Ricaud et al., 2007), resulted in more roots with EdU stain in the lowest columella root cap layer (Figures 2F–I), suggesting faster cell cycle progression and stem cell division. Thus, EdU staining reveals a role for ATR, ATM, and WEE1 in controlling decisive checkpoints for stem cell maintenance in the columella root cap. Taken together, these results provide proof-of-concept demonstrations that EdU staining can be used as a complementary technology to Lugol’s staining, to study the dynamics of stem cell behavior that govern homeostasis.

### EdU and Lugol’s Staining can be Integrated to Dissect the Effects of Auxin on Stem Cell Homeostasis

**FIGURE 3:**
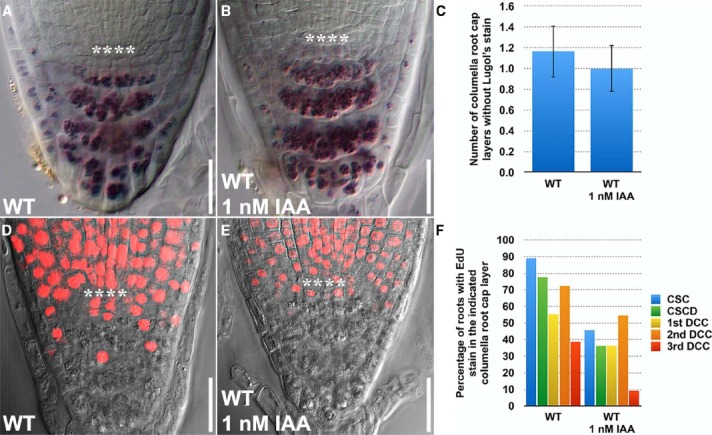
**EdU and Lugol’s staining can be combined and jointly used to investigate the effects of exogenously applied auxin on stem cell homeostasis in the *Arabidopsis* root. (A)** Lugol’s staining of WT (*n* = 14). **(B)** Lugol’s staining of WT treated with 1 nM IAA for 24 h (*n* = 13). **(C)** Quantification of the number of layers of unstained columella root cap cells. **(D)** EdU staining of WT treated with 10 μM EdU for 24 h (*n* = 18). **(E)** EdU staining of WT treated with 10 μM EdU and 1 nM IAA for 24 h (*n* = 13). **(F)** Quantification of the percentage of roots with EdU stain in the indicated columella root cap layer. Error bars represent standard error of the mean. **** represents position of the QC. CSC: layer of columella root cap stem cells; CSCD: layer of differentiating columella root cap stem cell daughters; 1st DCC: 1st layer of fully differentiated columella root cap cells; 2nd DCC: 2nd layer of fully differentiated columella root cap cells; 3rd DCC: 3rd layer of fully differentiated columella root cap cells. Scale bars: 20 μm.

Using the Lugol’s staining technology, auxin (1 μM NAA) was previously found to promote differentiation of columella root cap stem cells (Ding and Friml, 2010). However, it remains unclear how auxin influences stem cell division in the columella root cap. To address this, we next asked whether EdU and Lugol’s staining could be integrated to dissect the effects of auxin on stem cell division and differentiation, even when a very low concentration of auxin (1 nM IAA) was applied. With Lugol’s staining, we observed a slight decrease in the number of columella layers without Lugol’s staining (Figures 3A–C), indicating that the rate of differentiation was moderately accelerated by a small change in auxin concentration. Less of the IAA-treated seedlings had EdU stain in all the columella cell layers (Figures 3D–F), indicating that the rate of division of the columella stem cells were decreased. Hence, EdU staining, when used together with Lugol’s staining, provides a sensitive quantitative method to study the dynamics of stem cell behavior that govern homeostasis in the *Arabidopsis* columella root cap.

### EdU Staining Minimally Affects Root Growth within 24 h

**FIGURE 4:**
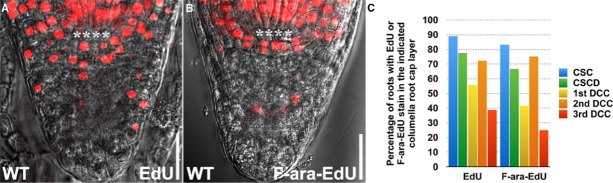
**Comparison of EdU and F-ara-EdU staining patterns in the columella root cap. (A)** WT treated with 10 μM EdU for 24 h (*n* = 18). **(B)** WT treated with 10 μM F-ara-EdU (*n* = 12) for 24 h. **(C)** Quantification of the percentage of roots with EdU or F-ara-EdU stain in the indicated columella root cap layer. **** represents position of the QC. CSC: layer of columella root cap stem cells; CSCD: layer of differentiating columella root cap stem cell daughters; 1st DCC: 1st layer of fully differentiated columella root cap cells; 2nd DCC: 2nd layer of fully differentiated columella root cap cells; 3rd DCC: 3rd layer of fully differentiated columella root cap cells. Scale bars: 20 μm.

Previously, (2′*S*)-2′-deoxy-2′-fluoro-5-ethynyluridine (F-ara-EdU), an arabinofuranosyl-ethynyluracil derivative, has been described as being less toxic to animal and plant cells (Neef and Luedtke, 2011; Pastrana, 2012; Cruz-Ramirez et al., 2013). We thus compared the staining patterns of EdU (Figure 4A) with F-ara-EdU (Figure 4B) in the columella root cap and found no obvious difference (Figure 4C). This suggests that the purported toxicity of EdU has minimal effect on the growth of *Arabidopsis* roots in 24 h. In fact, we found that much higher laser intensity is needed to capture the F-ara-EdU stain in roots, which could lead to false positives during imaging and quantification. Nonetheless, F-ara-EdU may still be useful for the analysis of cell division over long durations exceeding 24 h as has been reported (Neef and Luedtke, 2011; Cruz-Ramirez et al., 2013).

## Discussion

In this study, we have used EdU staining to identify dividing columella root cap stem cells and their progeny. This allows us to quantitatively analyze stem cell homeostasis in the columella root cap when combined with Lugol’s staining. Our method allows a better representation of columella stem cell homeostasis as compared to the conventional use of only Lugol’s staining. Using our method, we are able to pinpoint whether division or differentiation is disrupted, which cannot be distinguished by using the Lugol’s staining technology alone. Furthermore, EdU staining can also be used in mutants or transgenic plants with compromised starch metabolism, for which Lugol’s staining is not reliable. An additional advantage of EdU staining is that it can be used in conjugation with immunostaining, which allows for cell proliferation and other cellular events to be analyzed simultaneously (Zeng et al., 2010).

Given that EdU and Lugol’s staining are applicable to the columella root cap of other plant species such as rice (Wang et al., 2014), we anticipate that our method will be adopted widely to facilitate the dissection of stem cell homeostasis in plants.

## Author Contributions

JHH and JX designed the study. JHH developed protocols, performed experiments and imaged all the figures. HC optimized steps/methods, experimented on and refined imaging techniques and procedures and troubleshot errors to problems encountered during the experiments. ZC and DG contributed to solutions for problems (troubleshooting) they encountered and helped in improving some of the steps. XG contributed to optimization of fixation steps. JHH and JX analyzed the data and wrote the manuscript. All the authors commented and agreed on the manuscript.

### Conflict of Interest Statement

The authors declare that the research was conducted in the absence of any commercial or financial relationships that could be construed as a potential conflict of interest.
